# Identifying Solitary Granulomatous Nodules from Solid Lung Adenocarcinoma: Exploring Robust Image Features with Cross-Domain Transfer Learning

**DOI:** 10.3390/cancers15030892

**Published:** 2023-01-31

**Authors:** Bao Feng, Xiangmeng Chen, Yehang Chen, Tianyou Yu, Xiaobei Duan, Kunfeng Liu, Kunwei Li, Zaiyi Liu, Huan Lin, Sheng Li, Xiaodong Chen, Yuting Ke, Zhi Li, Enming Cui, Wansheng Long, Xueguo Liu

**Affiliations:** 1 Department of Radiology, Jiangmen Central Hospital, Jiangmen 529000, China; 2 School of Electronic Information and Automation, Guilin University of Aerospace Technology, Guilin 541004, China; 3 School of Automation Science and Engineering, South China University of Technology, Guangzhou 510641, China; 4 Department of Nuclear Medicine, Jiangmen Central Hospital, Jiangmen 529000, China; 5 Department of Radiology, Sun Yat-sen University Cancer Center, Guangzhou 510060, China; 6 Department of Radiology, Fifth Affiliated Hospital Sun Yat-sen University, Zhuhai 519000, China; 7 Department of Radiology, Guangdong Provincial People’s Hospital, Guangdong Academy of Medical Sciences, Guangzhou 510080, China; 8 Department of Radiology, Affiliated Hospital of Guangdong Medical University, Zhanjiang 524000, China; 9 Department of Radiology, The Seventh Affiliated Hospital of Sun Yat-sen University, Shenzhen 518000, China

**Keywords:** lung granulomatous nodule, lung adenocarcinoma, solitary pulmonary solid nodules, adaptive cross-domain transfer learning, whole slide image

## Abstract

**Simple Summary:**

This retrospective study aimed to find suitable source domain data in cross-domain transfer learning to extract robust image features and build a model to preoperatively distinguish LGN from LAC in SPSNs. The experiment showed that, compared with other source domains (such as ImageNet and LIDC), the transfer learning signature based on lung whole slide images as the source domain could extract more robust features (Wasserstein distance: 1.7108). Finally, a cross-domain transfer learning radiomics model combining transfer learning signatures based on lung whole slide images as the source domain, clinical factors and subjective CT findings was constructed. According to the validation cohort results of five centres (AUC range: 0.9074–0.9442), the cross-domain transfer learning radiomics model that combined multimodal data could assist physicians in preoperatively differentiating LGN from LAC in SPSNs.

**Abstract:**

Purpose: This study aimed to find suitable source domain data in cross-domain transfer learning to extract robust image features. Then, a model was built to preoperatively distinguish lung granulomatous nodules (LGNs) from lung adenocarcinoma (LAC) in solitary pulmonary solid nodules (SPSNs). Methods: Data from 841 patients with SPSNs from five centres were collected retrospectively. First, adaptive cross-domain transfer learning was used to construct transfer learning signatures (TLS) under different source domain data and conduct a comparative analysis. The Wasserstein distance was used to assess the similarity between the source domain and target domain data in cross-domain transfer learning. Second, a cross-domain transfer learning radiomics model (TLRM) combining the best performing TLS, clinical factors and subjective CT findings was constructed. Finally, the performance of the model was validated through multicentre validation cohorts. Results: Relative to other source domain data, TLS based on lung whole slide images as source domain data (TLS-LW) had the best performance in all validation cohorts (AUC range: 0.8228–0.8984). Meanwhile, the Wasserstein distance of TLS-LW was 1.7108, which was minimal. Finally, TLS-LW, age, spiculated sign and lobulated shape were used to build the TLRM. In all validation cohorts, The AUC ranges were 0.9074–0.9442. Compared with other models, decision curve analysis and integrated discrimination improvement showed that TLRM had better performance. Conclusions: The TLRM could assist physicians in preoperatively differentiating LGN from LAC in SPSNs. Furthermore, compared with other images, cross-domain transfer learning can extract robust image features when using lung whole slide images as source domain data and has a better effect.

## 1. Introduction

The detection rate of solitary pulmonary solid nodules (SPSNs) has greatly improved with the popularization of CT [1]. Lung adenocarcinoma (LAC) is the most common pathological type of malignant SPSN [2,3]. In contrast, lung granulomatous nodules (LGNs) are one of the great radiological mimickers of lung cancer and that are a common infectious disease causing serious medical and social problems [4,5]. LGN presenting as SPSNs has atypical imaging features, such as lobulated shape, spiculated sign and other subjective CT signs consistent with LAC, which brings difficulties for diagnostics [6,7]. It has been reported that the false-positive rate of LGN is in the range of 57.1% to 92.0% [8]. Previous studies have shown that percutaneous needle biopsy has high diagnostic value in the diagnosis of lung nodules, as invasive tissue sampling approaches are often selected based on the location of the nodule, comorbidities and the physical condition of patients. However, needle biopsy is associated with a risk for pneumothorax and haemorrhage [9,10]. Thus, it is of great value to develop an effective preoperative diagnosis method for the malignant risk of SPSNs. For LAC patients, a more active plan should be used for early diagnosis and to improve prognoses. Meanwhile, for LGN patients, unnecessary invasive procedures (such as needle biopsy or surgery) should be avoided because of various limitations, including cost, training expertise and potential for serious complications, such as pneumothorax and haemorrhage [3].

In recent years, artificial intelligence (AI) techniques coupled with radiological imaging have played an essential role in automatically predicting the nature of tumours [11]. Multivariate logistic regression analyses were applied to identify independent predictors of LGN and LAC from clinical characteristics and CT morphological features of lesions and to construct a model [8]. The CT morphological features of the lesions were obtained by two experienced chest radiologists. However, interreader variability with respect to manual nodule size measurement and visual assessment of radiologic features has been reported, which could lead to misdiagnoses [7]. Furthermore, Zhou et al. and Yang et al. created a radiomics nomogram combined with clinical features, CT morphological features of lesions and radiomics signature to differentiate LAC from LGN in patients with pulmonary solitary solid nodules using multivariate logistic regression analyses, respectively [9,12]. The radiomics features based on fixed calculation formulas were extracted from each three-dimensional lung nodule on thin-slice CT images, and radiomics signatures were built using least absolute shrinkage and selection operator logistic regression. However, radiomics also relies on precise tumour boundary annotation, which requires manual labelling and many human resources. In addition, radiomics features, where predesigned features are extracted from a region of interest, lack the specificity and sensitivity required to differentiate LGN from LAC in patients with SPSNs.

In contrast, advanced artificial intelligence models can overcome these problems through self-learning strategies, such as convolutional neural networks (CNNs). Deep learning features extracted using hierarchical convolution operations from the raw medical image will contain more abstract information about the lesion and may provide greater predictive insights [10]. CNN models have shown promising performance in assisting lung cancer analysis [13,14,15]. However, due to the capacity of CNNs to fit a wide diversity of nonlinear data points, they require a large amount of training data. This often makes CNNs prone to overfitting on small datasets, where the model tends to fit well to the training data but is not predictive for new data.

CNN based on transfer learning has been widely used because it does not require a precise delineation of lesions and can automatically extract features related to the target task in the case of small data [16,17]. Transfer learning seeks to transfer knowledge from predefined source domain data to a new target task [18]. In the field of medical image research of pulmonary nodules, the most widely used cross-domain transfer learning strategy is pretraining with fine-tuning: First, a source network is trained with a large source domain dataset (e.g., ImageNet: containing 1.3 million images, such as cats, dogs and flowers); second, the target network is initialized using learned weights of the source network; finally, the target domain data are used to fine-tune the target network [16,19].

However, the source domain data have a certain influence on the effect of cross-domain transfer learning. In particular, transfer learning based on fine-tuning is easily introduces redundant features when the source domain data are quite different from the target domain data, which leads to negative transfer and overfitting [20,21,22]. Therefore, eliminating redundant features in source domain data and adaptively selecting useful features for target task learning to constrain the training of the target network are crucial for cross-domain transfer learning [23].

To eliminate the adverse effects of redundant features in the source network on the target model, an adaptive source domain feature selection network in cross-domain transfer learning was used to select the features of the source network that are conducive to the learning of the target network to constrain the training of the target model. Then, we investigated the impact of the cross-domain transfer learning signature (TLS) based on different source domain data (e.g., ImageNet, lung whole slide images (WSIs) and CT images of the lung) on distinguishing LGN from LAC in SPSNs. In addition, a metric was introduced to measure the cross-domain transfer learning value of different source domain data to the target task. This technique was first applied to the preoperative differential diagnosis of LAC and LGN with SPSNs. Finally, a cross-domain transfer learning radiomics model (TLRM) combining TLS, clinical factors and subjective CT findings was constructed to assist clinicians in the preoperative diagnosis of LAC and LGN with SPSNs. Multicentre data were used for verification.

## 2. Materials and Methods

This study was approved by the institutional review board. The need for informed consent was waived because this was a retrospective study using preexisting imaging data.

### 2.1. Patients

**Figure 1:**
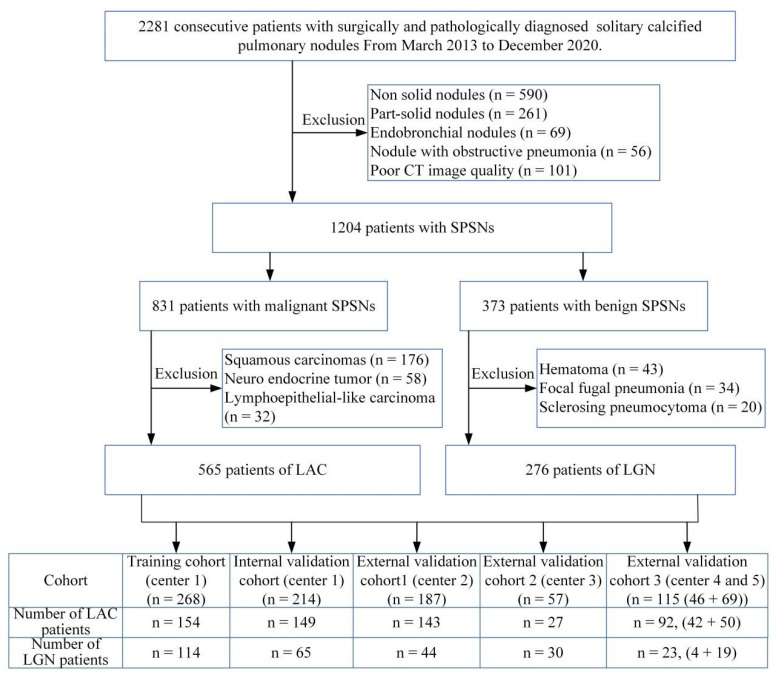
Flow diagram of the patient inclusion and exclusion process. Note: CT, computed tomography; SPSNs, solitary pulmonary solid nodules; LAC, lung adenocarcinoma; LGN, lung granulomatous nodule.

The enrolled SPSN patients with complete medical information and CT images were collected from five medical centres from March 2013 to December 2020. The inclusion criteria were as follows: (1) radical surgical resected SPSNs with final histopathological diagnosis confirmed LAC and LGN; (2) the diameter of the SPSNs ≤ 30 mm; (3) primary thoracic CT images with slice thickness 0.625-3.0 mm in the axial section; and (4) interval between preoperative thoracic CT examination and operation of less than 1 month. The exclusion criteria were as follows: (1) calcified nodules or solid nodules with a satellited patchy opacity that represented chronic inflammatory disease; (2) subsolid nodules in the nodule attenuation subtype; (3) thoracic CT images with artifacts that did not meet the diagnostic requirements; and (4) patients with a malignant tumour history. The flowchart of participants is shown in Figure 1. The pathological evaluation is shown in Supplementary S1.

Finally, 841 patients were included and divided into a training cohort that was used to train the model and four validation cohorts that were used to assess the performance of the models (Table 1).

In addition, to study the impact of source domain data on cross-domain transfer learning, the WSIs of lung cancer from the Cancer Genome Atlas (https://portal.gdc.cancer.gov, accessed on 11 October 2020), ImageNet (https://www.image-net.org, accessed on 3 October 2020) and CT images of pulmonary nodules from LIDC-IDRI (https://wiki.cancerimagingarchive.net/display/Public/LIDC-IDRI, 7 October 2020) were collected and separately used as source domain data for cross-domain transfer learning.

All images were preprocessed into three-channel images of 224 × 224 to meet the input requirements of the cross-domain transfer learning model (Supplementary S2 and Figure S1).

### 2.2. CT Scanning Parameters

The CT scanning parameters were as follows: 16-detector-row CT scanner and dual-energy Somatom Flash (Siemens Medical Systems, Forchheim, Germany), 64-detector-row CT scanner Aquilion One (Toshiba Medical Systems, Otawara, Japan), and 64-detector-row CT scanner GE Discovery (GE Healthcare, Boston, MA, USA). The scanned direction was caudocranial with the patient in the supine position. The scanned filed was from the bilateral lung tip to base with deep inhalation breath holding. Scanned parameters: tube voltage, 120 kVp; automated mAs technique; collimation, 16 × 0.75 mm or 64 × 0.5 mm; pitch 0.875–1.5; and matrix, 512 × 512. Primary axial CT images were obtained in standard (B40f) and high resolution (B70f) algorithms with slice thicknesses of 0.625–3.0 mm and coronal and sagittal planner images with slice thickness of 3.0 mm were reconstructed in the postprocess workstation.

### 2.3. Evaluation of Subjective CT Findings

Thoracic CT images were reviewed and recorded by two experienced radiologists from centre one (one with 12 years’ and another with 25 years’ experience in thoracic radiology); both were blinded to the medical information and pathological results. Agreement was reached through consultation when different opinions occurred. Thoracic CT images of each patient were reviewed in the radiologist workstation using a lung window (width, 1500 HU (Housfield); level, −600 HU) and mediastinal window (width, 300 HU; level, 40 HU). Radiological CT manifestations were recorded according to the glossary of terms for thoracic imaging by the Fleischner Society were as follows: (1) location; (2) diameter; (3) regular margin (presence or absence); (4) lobulated shape (presence or absence); and (5) spiculated sign (presence or absence) [24,25,26].

### 2.4. Building the Transfer Learning Signature (TLS)

#### 2.4.1. Transfer Learning Feature Extraction Based on an Adaptive Cross-Domain Transfer Learning Model

**Figure 2:**
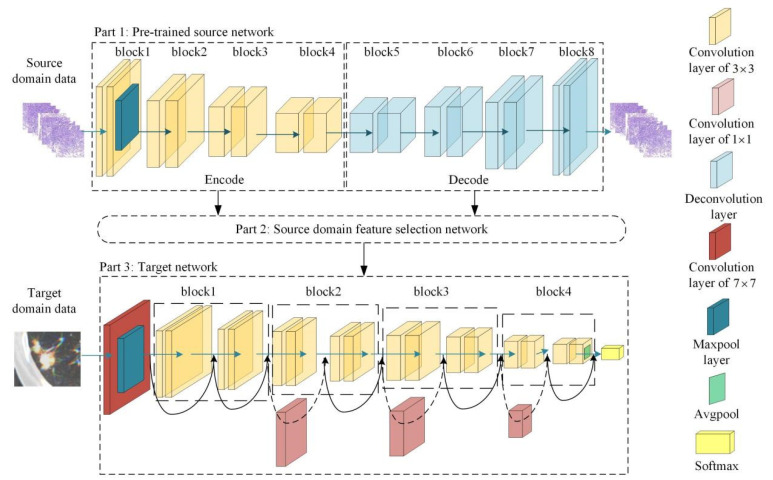
The structure of the adaptive cross-domain transfer learning model. The model has three parts: a pretrained source network; a target network; and a source domain feature selection network.

An adaptive cross-domain transfer learning model was used to extract the robust transfer learning features of SPSNs from CT images. As shown in Figure 2, this model has three parts: a pretrained source network, a target network and a source domain feature selection network. First, the pretrained source network was trained using source domain data to construct an intermediate feature space. Then, a source domain feature selection network was proposed. In the source domain feature selection network, two meta-networks were proposed to eliminate redundant features in source domain data and adaptively select useful features for target task learning to constrain the training of the target network in the constructed feature space. Finally, under the constraint of beneficial features, the target network was trained using CT images to obtain task-related robust transfer learning features. More details of the adaptive cross-domain transfer learning model are provided in Supplementary S3 and Figure S2.

When the target network was well trained, the convolution kernels of the target network were used as feature extractors to extract transfer learning features from the CT images of SPSNs (Figure S3). Finally, 3904 transfer learning features were extracted for each patient.

#### 2.4.2. Building the TLS Based on Transfer Learning Features

First, the Mann-Whitney U test was used to select transfer learning features that were significantly different. Second, the sparse Bayesian extreme learning machine (Supplementary S4 and Figure S4) was proposed to select features related to the target task and to build TLS [27].

#### 2.4.3. TLS Comparison Based on Different Source Domain Data

To comprehensively evaluate the TLS under different source domain data, four TLS were constructed and compared to each other. These TLS were based on lung cancer WSIs (TLS-LW), ImageNet (TLS-ImageNet) and LIDC-IDRI (TLS-LIDC) as source domain data. In addition, to evaluate the effect of cross-domain transfer learning, TLS under different source domain data were compared with a nontransfer learning signature (Non-TLS). For Non-TLS, we only trained the target network using CT images of the training cohort to extract features without pretraining.

### 2.5. Building the TLRM

#### 2.5.1. Building the TLRM

To comprehensively analyse patient information, the clinical factors (including gender and age), subjective CT findings (including size, location, margin, lobulated shape, spiculated sign) and the best performing TLS were combined to build the TLRM. First, the Cohen’s kappa test was used to analyse interreader agreements (Reader 1 and Reader 2) of subjective CT findings. Second, the Wilcoxon rank-sum test, Pearson’s chi-square test or Fisher’s exact test were performed to identify significantly different features. Third, the sparse Bayes-based least absolute shrinkage and selection operator (Supplementary S4) was used to select features with independent risk factors and build the TLRM.

#### 2.5.2. TLRM Evaluation and Comparison

To comprehensively evaluate the TLRM under a multicentre scenario, we compared the TLRM with two other methods: (1) a clinical model combining clinical factors and subjective CT findings (Supplementary S5), and (2) the best performing TLS. Furthermore, the TLRM was calibrated by performing calibration curve analysis. Stratification analyses of patient characteristics and CT scan protocols were carried out to evaluate the generalizability of the TLRM.

### 2.6. Prospective Clinical Validation

To further validate the performance of the model, a prospective validation cohort of 99 cases from medical centre 1 between January 2021 and December 2021 was collected to evaluate the robustness of the model.

### 2.7. Model Evaluation Index

The receiver operating characteristic curve, area under the curve (AUC), sensitivity, specificity, accuracy, positive probability value (PPV), and negative probability value (NPV) were calculated to evaluate the performance of the models. The DeLong test was used to evaluate significant differences between the AUCs of the models.

The Wasserstein distance between the source domain data and target domain data was calculated for each cross-domain transfer learning model to assess the similarity of the distribution between the two-domain data. The integrated discrimination improvement (IDI) was used to evaluate whether the new model could outperform the old model. Decision curve analysis was used to calculate the net benefit of the clinical utility of the model. All statistical analyses were two-tailed. A *p* value < 0.05 was statistically significant.

## 3. Results

### 3.1. Clinical Factors and Subjective CT Findings Analysis

The demographics and subjective CT findings of all cohort data are presented in Table 2. In the training cohort, the nodule size, nodule margin, lobulated shape, and spiculated sign showed good interobserver agreements (k = 0.837, 0.735, 0.743, and 0.755, respectively). The LAC and LGN groups differed significantly in characteristics, including gender, age, nodule size, nodule margin, lobulated shape, and spiculated sign (*p* < 0.05).

### 3.2. Comparison and Selection of TLS Based on Different Source Domain Data

#### 3.2.1. TLS Based on Different Source Domain Data vs. Non-TLS

The model details of TLS-LW, TLS-ImageNet, TLS-LIDC and Non-TLS are shown in Table S1. The results of these models are shown in Table 3 and Figure S5. In the whole validation data, the AUCs of TLS-LW, TLS-ImageNet, TLS-LIDC and Non-TLS were 0.8395, 0.7755, 0.7030, and 0.7156, respectively. Furthermore, the Delong test and IDI indicated that TLS-LW had significantly better predictive performance than Non-TLS in whole validation data (Delong test: *p* < 0.05; IDI = 0.0312 (*p* < 0.05), Table S2).

#### 3.2.2. Comparison and Selection of TLS Based on Different Source Domain Data

The scores of TLS-LW, TLS-ImageNet, TLS-LIDC and Non-TLS in the whole validation cohort are shown in Figure S6. The Delong test and IDI indicated that TLS-LW had significantly better predictive performance than TLS-ImageNet and TLS-LIDC in whole validation data. The *p* values of the DeLong test were all less than 0.05, and the IDI values were 0.0162 and 0.0341, with *p* values were all less than 0.05 (Table S2). Therefore, the TLS-LW was selected to build the TLRM.

**Figure 3:**
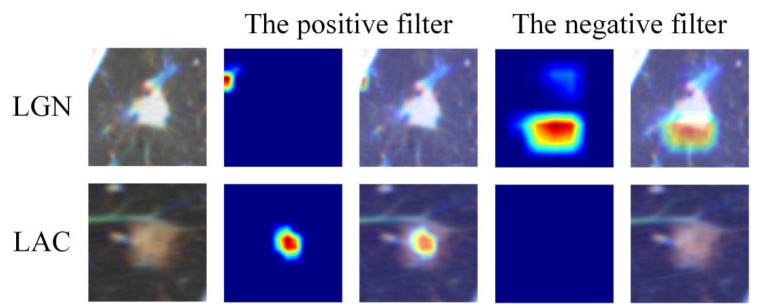
Visualization of two patient samples for TLS-LW. In the second and fourth columns, the heatmaps of the two convolution kernels are noted for two patients. In the third and fifth columns, the combination maps of two convolution kernel heatmaps and input data are noted for two patients. The red region represents a larger weight, which shows that the model focuses on the area of the CT image. Note: TLS-LW, transfer learning signature based on lung cancer WSI; LAC, lung adenocarcinoma; LGN, lung granulomatous nodule.

Tumours with different statuses can activate different signaling pathways in the model and can be encoded into different valued features. To explore the association between transfer learning features and lesion images, two lesion images from two patients (one LGN and one LAC) were fed into TLS-LW, and different responses were observed (Figure 3). The positive filter had strong responses to lesions of patients with LAC and weak responses to lesions of patients with LGN. The negative filter had strong responses to lesions of patients with LGN and was nearly shut down in lesions of patients with LAC. The visualization method is shown in Supplementary S6. In addition, the predicted value of TLS-LW revealed a significant difference between the LGN and LAC groups in all cohort data (*p* < 0.05; Table 2).

Furthermore, the Wasserstein distance was used to assess the similarity between the source domain and target domain data. A smaller Wasserstein distance represented a more similar distribution between the two-domain data [28]. The specific definition of the Wasserstein distance is shown in Supplementary S8. The distances of TLS-LW, TLS-ImageNet, and TLS-LIDC were 1.7108, 3.3567 and 3.6323, respectively. The Wasserstein distance of TLS-LW was minimal. In contrast, TLS-LIDC had the largest Wasserstein distance.

### 3.3. TLRM Construction and Evaluation

#### 3.3.1. TLRM Construction

**Figure 4:**
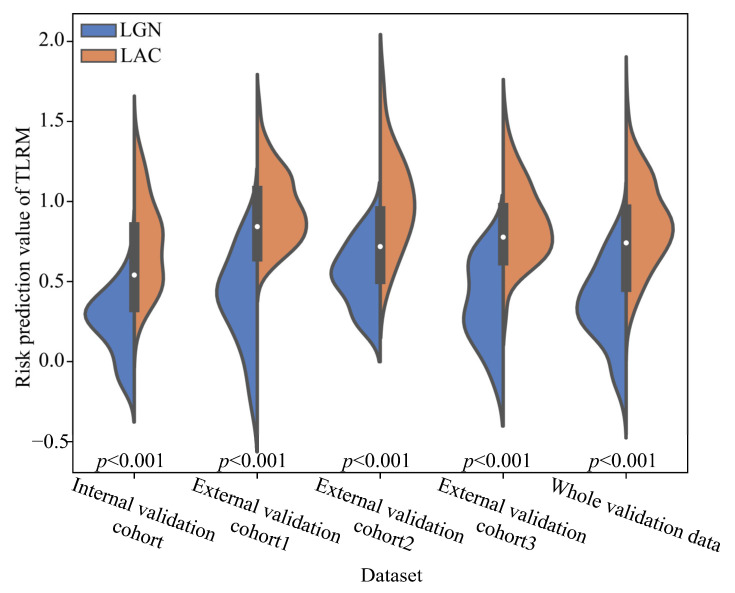
The risk prediction value of distributions of TLRM in the four validation cohorts and whole validation data. Note: TLRM, transfer learning radiomics model; LAC, lung adenocarcinoma; LGN, lung granulomatous nodule.

**Figure 5:**
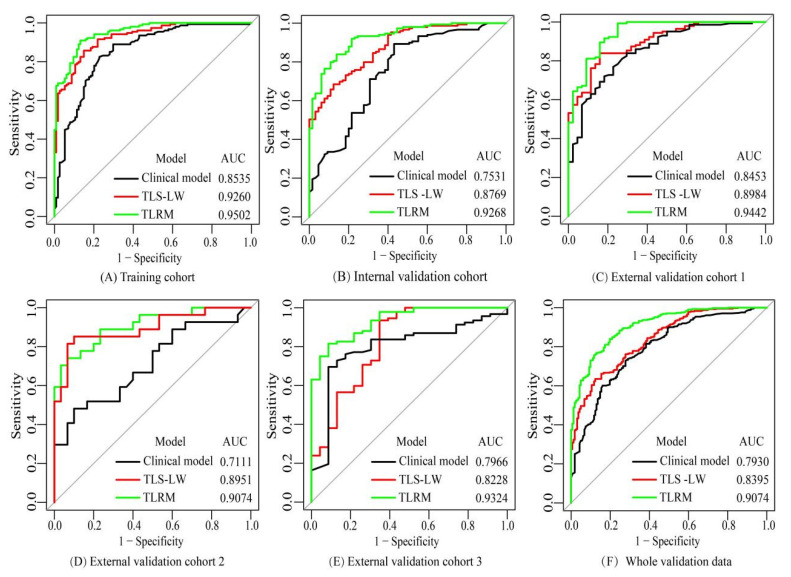
The receiver operating characteristic curves of the clinical model, TLS-LW, and TLRM in the training cohort (**A**), internal validation cohort (**B**), external validation cohort 1 (**C**), external validation cohort 2 (**D**), external validation cohort 3 (**E**) and whole validation data (**F**). Note: TLS-LW, transfer learning signature based on lung cancer WSI; TLRM, transfer learning radiomics model; AUC, area under the curve.

The results of the sparse Bayesian least absolute shrinkage and selection operator showed that age, lobulated shape, spiculated sign and TLS-LW were independent risk factors in distinguishing LGN and LAC lesions, and were used to develop the TLRM. The calculation formula of the risk prediction value based on the TLRM is shown in Supplementary S7. The risk prediction value of TLRM revealed a significant difference between the LGN and LAC groups in all validation cohorts (Figure 4). The AUCs of TLRM were 0.9268, 0.9442, 0.9074, 0.9324 and 0.9074 in the four validation cohorts and whole validation data, respectively (Table 4 and Figure 5).

**Figure 6:**
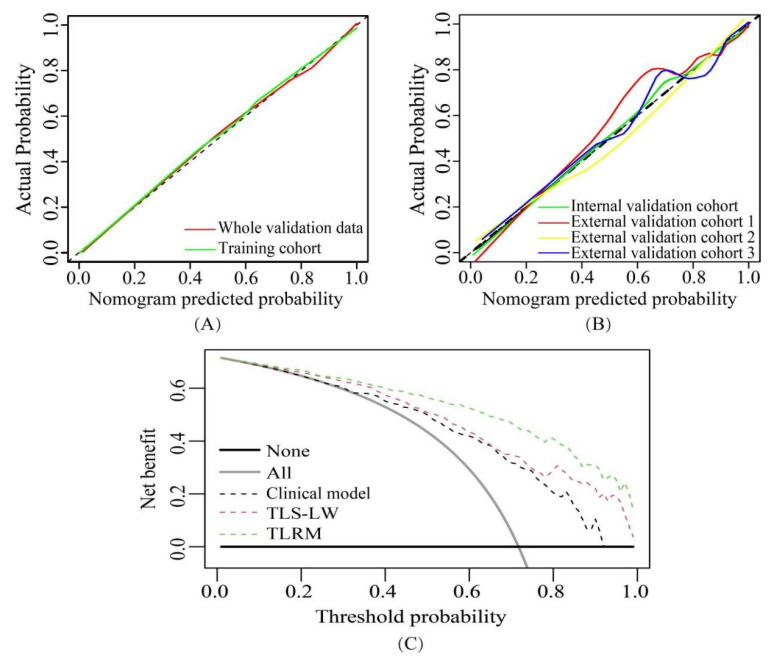
Calibration curves and decision curve analysis. (**A**) The calibration curves of TLRM in the training cohort and whole validation data. (**B**) The calibration curves of TLRM in the four validation cohorts. (**C**) Decision curve analysis of the clinical model, TLS-LW, and TLRM in whole validation data. The results showed that the net benefit of TLRM was greater than that of the clinical model and TLS-LW (range, 0.01–1.00). Note: TLS-LW, transfer learning signature based on lung cancer WSI; TLRM, transfer learning radiomics model.

The Hosmer-Lemeshow test yielded no significant difference between the predictive calibration curve and the ideal curve for risk status prediction in the four validation cohorts and whole validation data (Chi-square value range: 2.9224–4.9919, *p* value range: 0.7584–0.9391; Figure 6A,B)

#### 3.3.2. TLRM vs. TLS-LW and Clinical Model

The clinical model was built using multivariable logistic regression, gender, age, lobulated shape and spiculated sign were independent factors associated with LAC. The specific parameters of the clinical model are shown in Table S3.

The diagnostic performances of the clinical model, TLS-LW, and TLRM are shown in Table 4. The AUCs of the clinical model were 0. 7531, 0.8543, 0.7111, 0.7966 and 0.7930 in the four validation cohorts and whole validation data, respectively. The AUCs of TLS-LW were 0.8769, 0.8984, 0.8951, 0.8228 and 0.8395 in the four validation cohorts and whole validation data, respectively. The Delong test and IDI indicated that the TLRM had significantly better predictive performance than the clinical model and TLS-LW in the whole validation cohort (Delong test, *p* < 0.05; IDI = 0.0385 (*p* < 0.05), and Delong test, *p* < 0.05; IDI = 0.0222 (*p* < 0.05) Table S4).

Specifically, the false-positive rate of the clinical model was 28.40% (46/162) across whole validation data, while the false-positive rate of TLRM was only 17.90% (29/162), TLRM had the best effect.

#### 3.3.3. Stratified Analysis of TLRM

Using stratified analysis, the TLRM performance was found to be unaffected by the gender, age and image slice thickness of patients (Delong test, all *p* > 0.05). Stratification analysis was performed based on patient characteristics (gender and age) and CT slice thickness to evaluate the robustness of the TLRM. The AUC of TLRM in the overall cohort (*n* = 841) was 0.9248 (95% CI: 0.9071–0.9424).

Part 1: Stratified analysis of gender. The patients were divided into two groups: women (*n* = 409) and men (*n* = 432). The AUCs were 0.9301 (95% CI: 0.9042–0.9560) and 0.9167 (95% CI: 0.8914–0.9419), respectively, and the *p* values were 0.7382 and 0.6075, respectively, when the two groups and the overall cohort were compared using the DeLong test. The ROC curves are shown in Figure S7A.

Part 2: Stratified analysis of age. The patients were divided into two groups: age < 60 years (*n* = 433) and age ≥ 60 years (*n* = 408), with respective AUCs of 0.9229 (95% CI: 0.8987–0.9471) and 0.9182 (95% CI: 0.8899–0.9466). The *p* values were 0.9047 and 0.7014, respectively, when the two groups and overall cohort were compared using the DeLong test. The ROC curves are shown in Figure S7B.

Part 3: Stratified analysis of CT slice thickness. The patients were divided into two groups: thickness ≤ 1.5 mm (*n* = 536) and 1.5 < thickness ≤ 3 mm (*n* = 305), with AUCs of 0.9308 (95% CI 0.9091–0.9525) and 0.9091 (95% CI: 0.8772–0.9409), respectively. The *p* values were 0.6736 and 0.3978, respectively, when the two groups and overall cohort were compared using the DeLong test. The ROC curves are shown in Figure S7C.

The results showed that the characteristics of patients, and CT slice thickness had less impact on the stability and robustness of the proposed model.

#### 3.3.4. Clinical Use

The decision curve analysis indicated, in the threshold probability range of 0.01–1.00, a higher net benefit for TLRM in the whole validation data in differentiating the LAC and LGN groups than the clinical model and TLS-LW (Figure 6C).

### 3.4. Prospective Clinical Validation

**Figure 7:**
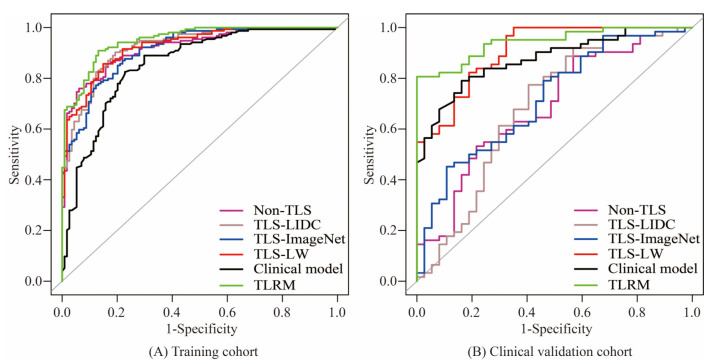
The receiver operating characteristic of Non-TLS, TLS-LIDC, TLS-ImageNet, clinical model, TLS-LW and TLRM in the training cohort (**A**) and clinical validation cohort (**B**). Note: Non-TLS, nontransfer learning signature; TLS-LIDC, transfer learning signature based on LIDC; TLS-ImageNet, transfer learning signature based on ImageNet; TLS-LW, transfer learning signature based on lung cancer WSI; TLRM, transfer learning radiomics model; AUC, area under the curve.

Demographic information and tumour characteristics of the prospective clinical validation cohort (*n* = 99) are listed in Table 1. When our TLS-LW was applied to a prospective clinical validation cohort (*n* = 99), the AUC was 0.9076, which was better than the results of Non-TLS, TLS-LIDC and TLS-ImageNet (Table 3 and Figure 7). Similarly, the AUC of TLRM in the prospective clinical validation set was 0.9429, which showed good performance (Table 4 and Figure 7).

## 4. Discussion

The diagnosis of LGN in patients with SPSNs can be difficult for clinicians since LGN shares some presupposed malignant morphological features with LAC. Currently, CNN is a promising tool in medical imaging research. However, it is prone to overfitting in the case of small data. In this retrospective study, we developed a TLRM based on adaptive cross-domain transfer learning in preoperatively distinguishing LGN from LAC in patients with SPSNs, which enabled early diagnosis and appropriate treatment for patients with LAC and minimization of unnecessary interventions and procedures for those with LGN.

Concerning the clinical factors, in the whole validation data, this study found that women were at higher risk for LAC, with 210 women with LAC and 60 women with LGN. The patients with LGN tended to be younger (mean age: 53.42 ± 11.99 years) than those with LAC (mean age: 60.13 ± 10.08 years), similar to previous studies [29,30]. Previous articles have shown that malignant nodules often manifest with irregular, spiculated and ill-defined margins whereas benign nodules have well-defined smooth edges [31]. Unfortunately, overlapping radiologic features based on CT images among LGN and LAC are inevitable phenomena. The lobulated shape histologically caused by chronic inflammatory cell infiltration and irregular interstitial fibrosis can also be seen in 25% of benign nodules [32]. Therefore, a significant proportion remains indeterminate, requiring follow-up or triggering invasive diagnostic procedures [33]. In our study, multivariate logistic regression analysis showed that age, gender, lobulated shape and spiculated sign were useful morphological features for differentiating LGN from LAC. However, the AUC and false-positive rate of the clinical model in the whole validation data were 0.7930 and 0.2840, respectively.

In recent years, some researchers have used artificial intelligence techniques coupled with radiological imaging to preoperatively differentiate LGN from LAC in SPSNs. Zhang et al. [8] selected age, sex and lobulated shape through multivariate logistic regression to build the model. The AUC of this model was as high as 0.956. However, this study only had 61 cases of data and was not a validation set. Yang et al. [12] constructed a combined radiomics model consisting of 19 radiomics features based on CT and five clinical risk factors. The AUCs of the combined radiomics in the training set (*n* = 221 cases) and validation test (*n* = 91 cases) were 0.92 and 0.84, respectively. Zhou et al. [9] constructed a radiomics nomogram consisting of six clinical features (spiculated sign, vacuole, minimum diameter of nodule, mediastinal lymphadenectasis, sex and age) and a radiomics signature based on 15 radiomics parameters. The AUCs of the radiomics nomogram in the training set (*n* = 220 cases) and validation test (*n* = 93 cases) were 1.00 and 0.99, respectively. Although combined radiomics and radiomics nomograms have shown good performance, their data are from a single centre and therefore cannot be used to evaluate model performance in the case of multicentre data. In addition, radiomics features, where predesigned features are extracted from a region of interest, lack the specificity and sensitivity required to differentiate LGN from LAC in patients with SPSNs.

CNN has excellent feature learning ability that can have specific features for tasks according to learning and does not require time-consuming tumour boundary annotations. However, CNNs are prone to overfitting on small datasets. In this study, we developed a Non-TLS. Unfortunately, the Non-TLS had the problem of overfitting (AUC of the training cohort: 0.9216, AUC of the whole validation data: 0.7156), perhaps because the training cohort data were relatively small when building the Non-TLS (3283 CT images).

Therefore, this study combined a convolutional neural network with cross-domain transfer learning. A source domain feature selection network was proposed to adaptively select features that were beneficial to target task learning to constrain training of the target network. In addition, we developed three cross-domain transfer learning signatures based on different source domain data: TLS-LW, TLS-LIDC and TLS-ImageNet. The results showed that the performances of TLS-LIDC and TLS-ImageNet were lower than that of TLS-LW. Compared with TLS-LIDC and TLS-ImageNet, the IDI of TLS-LW was 0.0341 (*p* < 0.05) and 0.0162 (*p* < 0.05) in the whole validation data, respectively. Interestingly, the Wasserstein distance of TLS-LW was minimal. In contrast, TLS-LIDC had the largest Wasserstein distance. These results indicate that for a small training dataset to truly take advantage of the transfer of learning the source domain data should be as similar as possible to the target domain data [20,34]. In addition, TLS-LW had better diagnostic performance than Non-TLS, and the IDI of TLS-LW was 0.0312 (*p* < 0.05) in whole validation data. The results showed that the transfer learning strategy helps to alleviate the overfitting problem of CNNs in the case of small samples.

Finally, age, lobulated shape, spiculated sign and TLS-LW were independent risk factors for distinguishing LGN and LAC lesions, which were used to develop TLRM. The decision curve analysis and IDI also indicated that TLRM had better performance than TLS-LW and the clinical model. Therefore, the TLRM combined with multimodal data has better diagnostic performance than image-based TLS alone and a clinical model based on clinical factors and subjective CT findings.

Despite encouraging results, the current study has several limitations. First, a selection bias may exist because of the retrospective nature of the study. Second, we only evaluated the difference between LAC and LGN, and other pathological types of lung nodules also need further investigation, such as lung squamous cell carcinoma, even metastatic lesions and other benign lesions [35]. Further work is needed to focus on incorporating other benign and malignant nodules into the classifier and validating it on a larger multisite dataset. Third, the chest CT images and WSIs of LAC came from different patients. The performance of diagnostic models based on CT images and WSIs of the matched patients needs to be further investigated.

## 5. Conclusions

The TLRM combined with multimodal data can assist physicians in preoperatively differentiating LGN and LAC presenting as SPSNs. We also found in this study that, compared with using other images as source domain data, cross-domain transfer learning has a better effect when using lung WSIs as source domain data.

## Figures and Tables

**Table 1:** Basic information of patients enrolled in the study.

	Training Cohort (*n* = 268)	Internal Validation Cohort (*n* = 214)	External Validation Cohort 1 (*n* = 187)	External Validation Cohort 2 (*n* = 57)	External Validation Cohort 3 (*n* = 115)	Whole Validation Data (*n* = 573)
CT scan time	January 2014 to May 2018	June 2018 to September 2020	January 2015 to December 2018	May 2018 to December 2020	March 2013 to August 2019	March 2013 to December 2020
**Pathological type**						
LGN	114	65	44	30	23	162
LAC	154	149	143	27	92	411
**Gender**						
Men	129	122	90	29	62	303
Women	139	92	97	28	53	270
**Age** **(mean ± SD, years)**	55.75 ± 12.49	58.54 ± 11.72	58.38 ± 10.78	56.37 ± 11.57	58.34 ± 9.98	58.23 ± 11.06
**Age range (years)**	20–81	16–79	28–80	31–79	27–75	16–80

Notes: CT, computed tomography; LAC, lung adenocarcinoma; LGN, lung granulomatous nodule; SD, standard deviation.

**Table 2:** Clinical factors and subjective CT findings from SPSNs in the LAC and LGN groups.

	**Training Cohort (*n* = 268)**			**Internal Validation Cohort (*n* = 214)**			**External Validation Cohort 1** **(*n* = 187)**		
	**LGN (114)**	**LAC (154)**	***p* Value**	**LGN (65)**	**LAC (149)**	***p* Value**	**LGN (44)**	**LAC (143)**	***p* Value**
**Gender**									
Men	64	65	0.0240 *	48	74	0.0010 *	28	62	0.0186 *
Women	50	89		17	75		16	81	
**Age (mean ± SD, years)**									
	50.96 ± 13.16	59.29 ± 10.70	<0.0001 ^#^	51.66 ± 12.65	61.54 ± 9.94	<0.0001 ^#^	56.20 ± 11.73	59.05 ± 10.31	0.1176 ^#^
**Nodule size (mean ± SD, mm)**									
	13.07 ± 8.00	17.31 ± 8.40	<0.0001 ^#^	12.05 ± 5.30	17.79 ± 8.57	<0.0001 ^#^	13.56 ± 8.11	21.74 ± 8.57	<0.0001 ^#^
**Location**									
LUL	27	44	0.0811 *	21	38	0.2682 *	10	35	0.6573 ^&^
LLL	14	33		5	28		8	22	
RUL	35	45		21	48		12	52	
RML	11	12		5	13		4	13	
RLL	27	20		13	22		10	21	
**Margin**									
Irregular	64	133	<0.0001 *	33	129	<0.0001 *	25	135	<0.0001 *
Regular	50	21		32	20		19	8	
**Lobulated shape**									
Absence	67	23	<0.0001 *	38	27	<0.0001 *	32	18	<0.0001*
Presence	47	131		27	122		12	125	
**Spiculated sign**									
Absence	100	74	<0.0001 *	53	83	0.0003 *	40	73	0.0005 *
Presence	14	80		12	66		4	70	
	**External Validation Cohort 2** **(*n* = 57)**			**External Validation Cohort 3** **(*n* = 115)**			**Whole Validation Data (*n* = 573)**		
	**LGN (30)**	**LAC (27)**	***p* Value**	**LGN (23)**	**LAC (92)**	***p* Value**	**LGN (162)**	**LAC (411)**	***p* Value**
**Gender**									
Men	14	15	0.5027 *	12	50	0.8516 *	102	201	0.0024 *
Women	16	12		11	42		60	210	
**Age (mean ± SD, years)**									
	51.53 ± 10.54	61.74 ± 10.37	<0.0001 ^#^	55.52 ± 10.99	59.04 ± 9.65	0.1436 ^#^	53.42 ± 11.99	60.13 ± 10.08	<0.0001 ^#^
**Nodule size (mean ± SD, mm)**									
	8.54 ± 4.07	18.14 ± 6.80	<0.0001 ^#^	16.52 ± 6.61	17.33 ± 5.56	0.3311 ^#^	12.45 ± 6.66	18.91 ± 7.65	<0.0001 ^#^
**Location**									
LUL	6	5	0.3856 ^&^	6	25	0.9844 ^&^	43	103	0.3856 *
LLL	5	5		4	12		22	67	
RUL	6	9		6	24		45	133	
RML	5	6		2	11		16	43	
RLL	8	2		5	20		36	65	
**Margin**									
Irregular	16	26	0.0002 *	19	82	0.4747 ^&^	93	372	<0.0001 *
Regular	14	1		4	10		69	39	
**Lobulated shape**									
Absence	20	8	0.0052 *	9	7	0.0005 ^&^	99	60	<0.0001 *
Presence	10	19		14	85		63	351	
**Spiculated sign**									
Absence	21	15	0.2590 *	19	28	<0.0001 *	133	199	<0.0001 *
Presence	9	12		4	64		29	212	
	**Clinical Validation Cohort (*n* = 99)**								
	**LGN (37)**						**LAC (62)**		***p* Value**
**Gender**									
Men	25						29		0.0444 *
Women	12						33		
**Age (mean ± SD, years)**									
	55.03 ± 10.32						60.76 ± 10.90		0.0103 ^#^
**Nodule size (mean ± SD, mm)**									
	12.73 ± 5.72						16.11 ± 6.16		0.0048 ^#^
**Location**									
LUL	6						13		0.9734 *
LLL	5						7		
RUL	13						20		
RML	3						6		
RLL	10						16		
**Margin**									
Irregular	21						52		0.0030 *
Regular	16						10		
**Lobulated shape**									
Absence	20						18		0.0133 *
Presence	17						44		
**Spiculated sign**									
Absence	30						6		<0.0001 *
Presence	7						56		

Notes: * Pearson’s chi-square test; ^&^ Fisher’s exact test; ^#^ Wilcoxon rank-sum test. CT computed tomography; SPSNs solitary pulmonary solid nodules; LAC lung adenocarcinoma; LGN lung granulomatous nodule; SD standard deviation; LUL left upper lobe; LLL left lower lobe; RUL right upper lobe; RML right middle lobe; RLL right lower lobe; TLS-LW transfer learning signature based on lung cancer WSI.

**Table 3:** Performance of transfer learning models based on different transfer sources (Non-TLS, TLS-LIDC, TLS-ImageNet, and TLS-LW) for the LAC and LGN groups among patients with SPSNs in the training cohort, four validation cohorts and whole validation data.

Dataset	Models	AUC (95% CI)	Sensitivity	Specificity	Accuracy	PPV	NPV
Training cohort (*n* = 268)	Non-TLS	0.9216 (0.8906–0.9526)	0.7597 (117/154)	0.9386 (107/114)	0.8358 (224/268)	0.9435 (117/124)	0.7431 (107/144)
	TLS-LIDC	0.9245 (0.8941–0.9549)	0.9026 (139/154)	0.8070 (92/114)	0.8619 (231/268)	0.8634 (139/161)	0.8598 (92/107)
	TLS-ImageNet	0.9136 (0.8814–0.9458)	0.8701 (134/154)	0.7807 (89/114)	0.8321 (223/268)	0.8428 (134/159)	0.8165 (89/109)
	TLS-LW	0.9260 (0.8964–0.9556)	0.8571 (132/154)	0.8509 (97/114)	0.8545 (229/268)	0.8859 (132/149)	0.8151 (97/119)
Internal validation cohort (*n* = 214)	Non-TLS	0.7949 (0.7295–0.8604)	0.7315 (109/149)	0.7846 (51/65)	0.7477 (160/214)	0.8862 (109/123)	0.5604 (51/91)
	TLS-LIDC	0.7740 (0.7048–0.8431)	0.6980 (104/149)	0.7385 (48/65)	0.7130 (152/214)	0.8595 (108/121)	0.5161 (48/93)
	TLS-ImageNet	0.8050 (0.7440–0.8659)	0.7450 (111/149)	0.7385 (48/65)	0.7430 (159/214)	0.8672 (111/128)	0.5581 (48/86)
	TLS-LW	0.8769 (0.8308–0.9231)	0.6846 (102/149)	0.8769 (57/65)	0.7430 (159/214)	0.9273 (102/110)	0.5481 (57/104)
External validation cohort 1 (*n* = 187)	Non-TLS	0.6465 (0.5458–0.7472)	0.9021 (129/143)	0.4091 (18/44)	0.7861 (147/187)	0.8323 (129/155)	0.5625 (18/32)
	TLS-LIDC	0.7972 (0.7195–0.8749)	0.8182 (117/143)	0.7045 (31/44)	0.7914 (148/187)	0.9000 (117/130)	0.5439 (31/57)
	TLS-ImageNet	0.7600 (0.6756–0.8445)	0.6084 (87/143)	0.8409 (37/44)	0.6631 (124/187)	0.9255 (87/94)	0.3987 (37/93)
	TLS-LW	0.8984 (0.8519–0.9450)	0.8392 (120/143)	0.8409 (37/44)	0.8396 (157/187)	0.9449 (120/127)	0.6167 (37/60)
External validation cohort 2 (*n* = 57)	Non-TLS	0.8123 (0.7029–0.9218)	0.7778 (21/27)	0.7667 (23/30)	0.7719 (44/57)	0.7500 (21/28)	0.7931 (23/29)
	TLS-LIDC	0.6778 (0.5390–0.8166)	0.6667 (18/27)	0.6333 (19/30)	0.6491 (37/57)	0.6207 (18/29)	0.6786 (19/28)
	TLS-ImageNet	0.7519 (0.6236–0.8801)	0.5556 (15/27)	0.8667 (26/30)	0.7193 (41/57)	0.7895 (15/19)	0.6842 (26/38)
	TLS-LW	0.8951 (0.8085–0.9816)	0.8519 (23/27)	0.9000 (27/30)	0.8772 (50/57)	0.8846 (23/26)	0.8710 (27/31)
External validation cohort 3 (*n* = 115)	Non-TLS	0.6541 (0.5276–0.7805)	0.8804 (81/92)	0.3913 (9/23)	0.7826 (90/115)	0.8526 (81/95)	0.4500 (9/20)
	TLS-LIDC	0.5416 (0.4257–0.6574)	0.2609 (24/92)	0.9565 (22/23)	0.4000 (46/115)	0.9600 (24/25)	0.2444 (22/90)
	TLS-ImageNet	0.7902 (0.6942–0.8862)	0.6196 (57/92)	0.8696 (20/23)	0.6696 (77/115)	0.9500 (57/60)	0.3636 (20/55)
	TLS-LW	0.8228 (0.7150–0.9306)	0.9348 (86/92)	0.6522 (15/23)	0.8783 (101/115)	0.9149 (86/94)	0.7143 (15/21)
Whole validation data (*n* = 573)	Non-TLS	0.7156 (0.6687–0.7625)	0.8273 (340/411)	0.6235 (101/162)	0.7696 (441/573)	0.8479 (340/401)	0.5872 (101/172)
	TLS-LIDC	0.7030 (0.6569–0.7491)	0.6399 (263/411)	0.7407 (120/162)	0.6684 (383/573)	0.8623 (263/305)	0.4478 (120/268)
	TLS-ImageNet	0.7755 (0.7374–0.8167)	0.6569 (270/411)	0.8086 (131/162)	0.6998 (401/573)	0.8970 (270/301)	0.4816 (131/272)
	TLS-LW	0.8395 (0.8058–0.8732)	0.8054 (331/411)	0.8395 (136/162)	0.8168 (468/573)	0.9272 (331/357)	0.6296 (136/216)
Clinical validation cohort (*n* = 99)	Non-TLS	0.6866 (0.5770–0.7961)	0.8871 (55/62)	0.4324 (16/37)	0.7172 (71/99)	0.7237 (55/76)	0.6957 (16/23)
	TLS-LIDC	0.6844 (0.5662–0.8026)	0.7742 (48/62)	0.5946 (22/37)	0.7071 (70/99)	0.7619 (48/63)	0.6111 (22/36)
	TLS-ImageNet	0.7162 (0.6118–0.8207)	0.4516 (28/62)	0.8919 (33/37)	0.6162 (61/99)	0.8750 (28/32)	0.4925 (33/67)
	TLS-LW	0.9076 (0.8503–0.9649)	1 (62/62)	0.6486 (24/37)	0.8687 (86/99)	0.8267 (62/75)	1 (24/24)

Notes: Numbers in parentheses were used to calculate percentages. SPSNs, solitary pulmonary solid nodules; LAC, lung adenocarcinoma; LGN, lung granulomatous nodule; CI, confidence interval; PPV, positive predictive value; NPV, negative predictive value; Non-TLS, nontransfer learning signature; TLS-LIDC, transfer learning signature based on LIDC; TLS-ImageNet, transfer learning signature based on ImageNet; TLS-LW, transfer learning signature based on lung whole slide images.

**Table 4:** Performance of the clinical model, TLS-LW, and TLRM for the LAC and LGN groups among patients with SPSNs in the training cohort, four validation cohorts and whole validation data.

Dataset	Models	AUC (95% CI)	Sensitivity	Specificity	Accuracy	PPV	NPV
Training cohort (*n* = 268)	Clinical model	0.8535 (0.8066–0.9004)	0.8247 (127/154)	0.7719 (88/114)	0.8022 (215/268)	0.8301 (127/153)	0.7652 (88/115)
	TLS-LW	0.9260 (0.8964–0.9556)	0.8571 (132/154)	0.8509 (97/114)	0.8545 (229/268)	0.8859 (132/149)	0.8151 (97/119)
	TLRM	0.9502 (0.9274–0.9731)	0.9091 (140/154)	0.8684 (99/114)	0.8918 (239/268)	0.9032 (140/155)	0.8761 (99/113)
Internal validation cohort (*n* = 214)	Clinical model	0.7531 (0.6798–0.8263)	0.8926 (133/149)	0.5692 (37/65)	0.7944 (170/214)	0.8261 (133/161)	0.6981 (37/53)
	TLS-LW	0.8769 (0.8308–0.9231)	0.6846 (102/149)	0.8769 (57/65)	0.7430 (159/214)	0.9273 (102/110)	0.5481 (57/104)
	TLRM	0.9268 (0.8923–0.9613)	0.9195 (137/149)	0.7846 (51/65)	0.8785 (188/214)	0.9073 (137/151)	0.8095 (51/63)
External validation cohort 1 (*n* = 187)	Clinical model	0.8543 (0.7926–0.9160)	0.8392 (120/143)	0.7045 (31/44)	0.8075 (151/187)	0.9023 (120/133)	0.5741 (31/54)
	TLS-LW	0.8984 (0.8519–0.9450)	0.8392 (120/143)	0.8409 (37/44)	0.8396 (157/187)	0.9449 (120/127)	0.6167 (37/60)
	TLRM	0.9442 (0.9067–0.9817)	0.9930 (142/143)	0.7500 (33/44)	0.9358 (175/187)	0.9281 (142/153)	0.9706 (33/34)
External validation cohort 2 (*n* = 57)	Clinical model	0.7111 (0.5755–0.8467)	0.4815 (13/27)	0.9000 (27/30)	0.7018 (40/57)	0.8125 (13/16)	0.6585 (27/41)
	TLS-LW	0.8951 (0.8085–0.9816)	0.8519 (23/27)	0.9000 (27/30)	0.8772 (50/57)	0.8846 (23/26)	0.8710 (27/31)
	TLRM	0.9074 (0.8314–0.9834)	0.7407 (20/27)	0.9333 (28/30)	0.8421 (48/57)	0.9091 (20/22)	0.8000 (28/35)
External validation cohort 3 (*n* = 115)	Clinical model	0.7966 (0.6962–0.8969)	0.6957 (64/92)	0.9130 (21/23)	0.7391 (85/115)	0.9697 (64/66)	0.4286 (21/49)
	TLS-LW	0.8228 (0.7150–0.9306)	0.9348 (86/92)	0.6522 (15/23)	0.8783 (101/115)	0.9149 (86/94)	0.7143 (15/21)
	TLRM	0.9324 (0.8839–0.9809)	0.8152 (75/92)	0.9130 (21/23)	0.8348 (96/115)	0.9740 (75/77)	0.5526 (21/38)
Whole validation data (*n* = 573)	Clinical model	0.7930 (0.7529–0.8332)	0.8029 (330/411)	0.7160 (116/162)	0.7784 (446/573)	0.8777 (330/376)	0.5888 (116/197)
	TLS-LW	0.8395 (0.8058–0.8732)	0.8054 (331/411)	0.8395 (136/162)	0.8168 (468/573)	0.9272 (331/357)	0.6296 (136/216)
	TLRM	0.9074 (0.8825–0.9324)	0.9100 (374/411)	0.8210 (133/162)	0.8848 (507/573)	0.9280 (374/403)	0.7824 (133/170)
Clinical validation cohort (*n* = 99)	Clinical model	0.8745 (0.8079–0.9410)	0.7903 (49/62)	0.8378 (31/37)	0.8081 (80/99)	0.8909 (49/55)	0.7045 (31/44)
	TLS-LW	0.9076 (0.8503–0.9649)	1 (62/62)	0.6486 (24/37)	0.8687 (86/99)	0.8267 (62/75)	1 (24/24)
	TLRM	0.9429 (0.9016–0.9842)	0.8065 (50/62)	1 (37/37)	0.8788 (87/99)	1 (50/50)	0.4551 (37/49)

Notes: Numbers in parentheses were used to calculate percentages. SPSNs, solitary pulmonary solid nodules; LAC, lung adenocarcinoma; LGN, lung granulomatous nodule; CI, confidence interval; PPV, positive predictive value; NPV, negative predictive value; TLS-LW, transfer learning signature based on lung whole slide images; TLRM, transfer learning radiomics model.

## Data Availability

The data presented in this study are available on request from the corresponding author. The data are not publicly available due to privacy restrictions.

## Supplementary Materials

The following supporting information can be downloaded at: https://www.mdpi.com/article/10.3390/cancers15030892/s1, References [36,37,38,39,40,41] are cited in the supplementary materials, Supplementary S1: Pathological evaluation; Supplementary S2: Image preprocessing; Supplementary S3: The adaptive transfer learning model; Supplementary S4: Sparse Bayes-based extreme learning machine (SB-ELM); Supplementary S5: Building the clinical model; Supplementary S6: Model visualization method; Supplementary S7: The calculation formula of risk prediction value of transfer learning radiomics model (TLRM); Supplementary S8: Wasserstein distance; Table S1: The model details of TLS-LW, TLS-ImageNet, TLS-LIDC and non-TLS; Table S2: The results of the TLS-LW were compared with the Non-TLS, TLS-LIDC, and TLS-ImageNet by Delong test and IDI; Table S3: Independent risk factors associated with LAC in clinical models by multivariate logistic regression; Table S4: The results of the TLRM were compared with those of the clinical model and TLS-LW by Delong test and IDI. Figure S1: The pre-processing processes of images. Figure S2: The structure of source domain feature selection networks. Figure S3: Processes of transfer learning feature extraction for each patient. Figure S4: Feature selection and structure of sparse Bayes-based extreme learning machine. Figure S5: ROC curves of Non-TLS, TLS-ImageNet, TLS-LIDC, and TLS-LW. Figure S6: The score of TLS-LW, TLS-ImageNet, TLS-LIDC and non-TLS in the whole validation data. Figure S7: Stratified analysis of gender, age, and CT slice thickness.
