# Supplementary material for: Identifying Solitary Granulomatous Nodules from Solid Lung Adenocarcinoma: Exploring Robust Image Features with Cross-Domain Transfer Learning
Source: Cancers (Basel). 2023 Jan 31;15(3):892. doi: 10.3390/cancers15030892 (PMC9913209; doi:10.3390/cancers15030892)
Supplement: Supplementary file 1 [file cancers-15-00892-s001.zip › cancers-2018073-supplementary.pdf]

Supplemental Materials

Supplementary S1: Pathological evaluation

All specimens were fixed with formalin and then routinely stained with hematoxylin and eosin (HE). All lesions were diagnosed and categorized in the basis of the 2011 International Association for the Study of Lung Cancer/the American Thoracic Society/the European Respiratory Society (IASLC/ATS/ERS) classification systems [36, 37].

Supplementary S2: Image preprocessing

For radiological CT images: as showed in **Figure S1A**, firstly, we cropped the CT images by finding a rectangular region of interest (ROI) that enclosed the outlined pulmonary nodules by reader 1. Secondly, we resized the pulmonary nodules patch to a 224 by 224 square. Thirdly, a series of three channel images which were composed of three consecutive slices. Finally, there were 3, 283 ROI image of three-channel of size 224 by 224 to train the target network. For LIDC-IDRI, there were 22, 593 CT images.

For pathological WSIs: as showed in **Figure S1B**, firstly, each WSI was divided equally into 400 small images. Because the original WSIs were too large. However, some of the small images were blank. Secondly, the blank images were discarded to eliminate the influence on model training. Thirdly, we resized the small images to a 224 by 224 square. At this time, these images were preprocessed as three-channel images of size 224 by 224. Finally, there were 301, 547 WSIs of LAC, and 274, 986 WSIs of LSC, respectively.

For nature images in ImageNet, we resized the images to a 224 by 224 square. Finally, there were 1, 300, 000 nature images of three-channel of size 224 by 224. (**Figure S1C**)

Supplementary S3: The adaptive transfer learning model

The structure of adaptive transfer learning model: The adaptive transfer learning model has three parts: pre-trained source network, target network and source domain feature selection network. The pre-trained source network was a auto encoder. The auto encoder included an encoder network and a decoder network. The encoder network included 4 convolution blocks that had 8 convolution layers. In addition, max-pooling was used between some convolutional layers to eliminate redundant features. Finally, the CT image was encoded into a 14 by 14 feature map. Similarly, the decoder network included 4 deconvolution blocks that have 8 deconvolution layers. The decoder network used the 14 by 14 feature map to reconstruct the original image. If the 14 by 14 feature map extracted the intrinsic characteristics of the image, the decoder network should be able to reconstruct the original images from the 14 by 14 feature map. Because the task of the auto encoder is to reconstruct the input image, only the image without label information were needed to train the auto encoder. The pre-trained source network was trained using source domain data.

The target network was a typical CNN that included 4 convolution blocks that has 17 convolution layers. The skip connection was used to alleviate the optimization difficulties caused by nonlinearity.

If pre-trained source network was well-trained on a source task, then its intermediate feature spaces should have useful knowledge for the task. Thus, mimicking the well-trained knowledge might be helpful for training another network. The source domain feature selection network was constructed to constrain the training of target network by selecting the useful features for target task in pre-trained source network. **Figure S2** provide an illustration of source domain feature selection network. In the source domain feature selection network, to achieve the goal that was then to train target network utilizing the knowledge of pre-trained source network, the feature matching loss was designed and minimized. Similar to that used in FitNet [38], this loss is represented by $L_{\text{wfm}}(\theta|x,\phi)$.

$$L_{\text{wfm}}(\theta|x,\phi) = \sum_{(m,n\in P)} \lambda^{m,n} \frac{1}{H\times W} \sum_c w_c^{m,n} \sum_{i,j} (r_\theta(T_\theta^n(x))_{c,i,j} - S^m(x)_{c,i,j})^2 \qquad (1)$$

Where x was the input of target network. $S^m(x)$ was intermediate feature maps of the mth block of the pre-trained source network. $T_\theta^n(x)$ was intermediate feature maps of the nth block of the target network with parameter θ. r_θ was a linear

transformation parameterized by θ such as a pointwise convolution. Here, the parameter θ consisted of both the parameter for linear-transformation r_θ and non-linear target network T_θ, where the former was only necessary in training the latter and was not required at testing time. $H \times W$ was the spatial size of $S^m(x)$ and $r_\theta(T_\theta^n(x)$, the inner-summation was over $i \in \{1,2,\cdots,H\}$ and $j \in \{1,2,\cdots,H\}$, and $w_c^{m,n}$ was the non-negative weight of channel c. $\lambda^{m,n}$ was the non-negative weight of transfer between the mth and nth blocks of source and target network, respectively.

Since the important channels to transfer can vary for each input image, the channel weights would be as a learnable function, $w^{m,n}=\left[w_c^{m,n}\right]=f_\phi^{m,n}(S^m(x))$, by taking the softmax output of a small meta-network which takes features of source models as an input. We let ϕ denote the parameters of meta-networks throughout this paper. When transferring knowledge from a source model to a target model, deciding pairs (m,n) of layers in the source and target model were crucial to its effectiveness. We also set $\lambda^{m,n}=g_\phi^{m,n}(S^m(x))$ for each pair (m,n) as an output of a meta-network $g^{m,n}$ that automatically decides important pairs of layers for learning the target task. Finally, we construct the meta-networks as 1-layer fully-connected networks for each pair $(m,n) \in P$ where P was the set of candidates of pairs. It takes the global average pooling features of the mth block of the pre-trained source network as an input, and outputs $w_c^{m,n}$ and $\lambda^{m,n}$. As for the channel assignments w, we used the softmax activation to generate them while satisfying $\sum_c w_c^{m,n}=1$, and for transfer amount λ between blocks, we commonly used ReLU6 [39] to ensure non-negativity of λ and to prevent $\lambda^{m,n}$ from becoming too large. N

So, final loss $L_{\text{total}}(\theta|x,y,\phi)$ to train a target network then is given as:

$$L_{\text{total}}(\theta|x,y,\phi) = L_{org}(\theta|x,y) + \beta L_{\text{wfm}}(\theta|x,\phi) \tag{2}$$

Where $L_{org}(\theta|x,y)$ is the original loss of target network (e.g., cross entropy) and $\beta > 0.$ is a hyper-parameter.

The training strategy of adaptive transfer learning model: the goal of model was to achieve high performance on the target task when the target network is learned using the training objective $L_{\text{total}}(\theta|x,y,\phi)$. To maximize the performance, the feature matching term $L_{\text{wfm}}(\theta|x,\phi)$ should encourage learning of useful features for the target

task. To measure and increase usefulness of the feature matching decided by meta-networks parameterized by ϕ, a four-stage training scheme was used [23].

In the first stage, given the current parameter $\theta_0 = \theta$, we updated the target network for T times via gradient-based algorithms for minimizing L_{wfm}. Namely, the result parameter θ_T was learned only using the knowledge of the pre-trained source network. Since transfer is done by the form of feature matching, it was feasible to train useful features for the target task by selectively mimic the source features. More importantly, it increased the influence of the regularization term L_{wfm} on the learning procedure of the target model in the inner-loop, since the target features were solely trained by the source knowledge (without target labels). The second stage was a one-step adaptation θ_{T+1} from θ_T toward the target labels. Then, in the third stage, the task-specific objective $L_{org}(\theta_{T+1}|x,y)$ can measure how quickly the target model had adapted (via only one step from θ_T) to the target task, under the sample used in the first and second stage. Finally, the meta-parameter ϕ can be trained by minimizing $L_{org}(\theta_{T+1}|x,y)$.

The training parameters of adaptive transfer learning model: Our method was implemented in Python 3.7 and performed on a machine with an Intel Core i9-9900K CPU and 64 GB memory. The model training was implemented using GPU-Torch and was accelerated on an NVIDIA RTX3090 (24 GB on-board memory).

All pretrained source networks were trained by stochastic gradient descent with a momentum of 0.9. We used a weight decay of 1e-4 and an initial learning rate of 0.1 and decayed the learning rate with cosine annealing. For all experiments, we pretrained source networks for 400 epochs. The size of the batch was 300 for small image experiments. The mean square error was used as the loss function.

All target networks were trained by stochastic gradient descent with a momentum of 0.9. We used a weight decay of 1e-4 and an initial learning rate of 0.1 and decayed the learning rate with cosine annealing. For all experiments, we trained target networks for 200 epochs. The size of the mini-batch was 300 for small image experiments. When using feature matching, we used $\beta = 0.5$. We used the Adam optimizer [40] for training the meta-networks f_ϕ , g_ϕ with a learning rate of 1e-3 or 1e-4 and a weight decay of 0 or 1e-4. In our meta-training scheme, we observed that T = 2 is sufficient to learn what and where to transfer."

Supplementary S4: Sparse Bayes-based extreme learning machine (SB-ELM)

As showed in **Figure S4**, the SB-ELM was a single layer feedforward neural network. The input weights ($W \in \mathbb{R}^{M \times L}$) of SB-ELM were randomly generated according to any continuous distribution function. M was the number of input features of each sample. L was the number of hidden layer neurons. The output weights of SB-ELM were analytically computed by sparse Bayesian based least absolute shrinkage and selection operator (SB-LASSO).

Then the objective function of SB-LASSO was

$$\hat{\mathrm{w}} = \arg\min \|t - HQ\|^2 + \lambda \sum_{i=1}^{L} \|\varphi_i\|_1 \qquad (3)$$

Here, $t \in \mathbb{R}^{N \times 1}$ was ground-truth class labels. N was the number of sample. $H \in \mathbb{R}^{N \times L}$ was the output of the hidden layer. $Q = (\varphi_1, \cdots \varphi_L) \in \mathbb{R}^{L \times 1}$ was output weights of sparse Bayes-based extreme learning machine. λ was a hyper-parameter.

The algorithm based on sparse Bayesian learning and automatic relevance determination was used to analytically compute unknown parameter [27]. It can automatically select features related to the target task for modelling, which can enhance the generalization performance of the SB-ELM.

Supplementary S5: Building the clinical model

The clinical model incorporating the clinical factors and subjective CT findings was built in three steps. Firstly, we analyzed inter-reader agreements (reader 1 vs reader 2) of subjective CT findings by using Cohen's kappa test. Secondly, statistical tests for between-group differences in the clinical characteristics (age and gender) and subjective CT findings were conducted using the Wilcoxon rank sum test and Pearson chi-square test. Thirdly, factors with statistically significant differences were selected to develop a clinical model by multivariate logistic regression with a stepwise forward selection of variables, according to Akaike's information.

Supplementary S6: Model visualization method

The model attention to the tumor was visualized by the convolutional filter visualization technique to explain the tumor information was learned by the model [41]. The image included in tumor lesion was inputted to the model and the activation reaction of the particular M filters was visualized by the following steps: (1) Exporting the output 2-D matrixes of particular filters; (2) Normalizing the values of the 2-D matrix to values that between 0-1 and resizing the 2-D matrix to the shape of input image, then converting 2-D matrix to grayscale map that the value is between 0-255; (3) Converting the grayscale map to RGB map. The response of filter to different type lesion was different, which helped to further prove the effectiveness of features.

Supplementary S7: The calculation formula of risk prediction value of transfer learning radiomics model (TLRM)

The risk prediction value of TLRM =

–0.0832
– 0.0783 × age
+ 0.2044 × lobulated shape
+ 0.1696 × spiculated sign
+ 0.8765 × TLS-LW score

Supplementary S8: Wasserstein distance

The Wasserstein distance was used to measure the similarity between the target images (CT image of SPSNs) and the reconstructed images by the pre-trained source network in adaptive transfer learning model. This was defined as following [28]:

$$W(P_1,P_2)=inf_{\gamma\sim S(P_1,P_2)}E(x,y)\gamma\left[\|x-y\|\right] \tag{4}$$

P_1 and P_2 were two images distributions, $S(P_2,P_2)$ was a set of all possible joint distributions combined with P_1 and P_2 distributions. For each possible joint distribution γ, sampling (x, y) ~ γ can get a sample of x and y, and calculated the distance of the sample $\|x-y\|$, so we can calculate the joint distribution, the sample's expectations of the distance $E(x,y)\sim\gamma\left[\|x-y\|\right]$.The lower bound on this expected value in all possible joint distributions $inf_{\gamma\sim S(P_1,P_2)}E(x,y)\gamma\left[\|x-y\|\right]$ was the Wasserstein distance.

Table S1. The model details of TLS-LW, TLS-ImageNet, TLS-LIDC and non-TLS.

Model	Total number of features	Number of features with $p < 0.05$ by Mann-Whitney U test	Number of neurons of input layer of ELM	Number of neurons of hidden layer of ELM	Number of selected neurons of hidden layer of ELM by SB-LASSO
Non-TLS	3904	209	209	207	37
TLS-LIDC	3904	32	32	161	31
TLS-ImageNet	3904	77	77	157	30
TLS-LW	3904	73	73	173	30

Notes: Non-TLS, non-transfer learning signature; TLS-LIDC, transfer learning signature based on LIDC; TLS-ImageNet, transfer learning signature based on ImageNet; TLS-LW, transfer learning signature based on lung whole slide images; ELM, extreme learning machine; SB-LASSO, sparse Bayesian based least absolute shrinkage and selection operator (SB-LASSO).

Table S2. The results of the TLS-LW were compared with the Non-TLS, TLS-LIDC, and TLS-ImageNet by Delong test and IDI.

DeLong test				
Dataset	Model 1 / Model 2	Non-TLS	TLS-LIDC	TLS-ImageNet
Internal validation cohort	TLS-LW	$p = 0.0215$	$p = 0.0070$	$p = 0.0110$
External validation cohort 1	TLS-LW	$p < 0.0005$	$p = 0.0094$	$p = 0.0027$
External validation cohort 2	TLS-LW	$p = 0.2007$	$p = 0.0091$	$p = 0.0527$
External validation cohort 3	TLS-LW	$p = 0.0359$	$p = 0.0026$	$p = 0.4583$
Whole validation data	TLS-LW	$p < 0.0005$	$p < 0.0005$	$p = 0.0015$

IDI				
Dataset	Model 1 / Model 2	Non-TLS	TLS-LIDC	TLS-ImageNet
Internal validation cohort	TLS-LW	0.0264 ($p = 0.0150$)	0.0245 ($p = 0.0290$)	0.0200 ($p = 0.0190$)
External validation cohort 1	TLS-LW	0.0354 ($p = 0.0492$)	0.0195 ($p = 0.0497$)	0.0208 ($p = 0.0923$)
External validation cohort 2	TLS-LW	0.0059 ($p = 0.7523$)	0.0517 ($p = 0.0042$)	0.0238 ($p = 0.1666$)
External validation cohort 3	TLS-LW	0.0560 ($p = 0.0049$)	0.0873 ($p = 0.0001$)	0.0205 ($p = 0.1151$)
Whole validation data	TLS-LW	0.0312 ($p = 0.0002$)	0.0341 ($p < 0.0005$)	0.0162 ($p = 0.0062$)

Notes: IDI, integrated discrimination improvement; Non-TLS, non-transfer learning signature; TLS-LIDC, transfer learning signature based on LIDC; TLS-ImageNet, transfer learning signature based on ImageNet; TLS-LW, transfer learning signature based on lung whole slide images.

Table S3. Independent risk factors associated with LAC in clinical models by multivariate logistic regression.

Factor	Weight	OR(95%CI)	*P value*
Gender	-0.879	0.415 (0.220-0.782)	$p = 0.006$
Age	0.047	1.048 (1.021-1.076)	$p = 0.001$
Lobulated shape	1.989	7.305 (3.701-14.418)	$p < 0.001$
Spiculated sign	1.892	6.630 (3.239-13.569)	$p < 0.001$
Constant	-3.743		

Notes: LAC, lung adenocarcinoma; CI, confidence interval; OR, odds ratio.

Table S4. The results of the TLRM were compared with those of the clinical model and TLS-LW by Delong test and IDI.

Delong test			
Dataset	Model 1 / Model 2	Clinical model	TLS-LW
Internal validation cohort	TLRM	$p < 0.0005$	$p = 0.0209$
External validation cohort 1	TLRM	$p < 0.0005$	$p = 0.0139$
External validation cohort 2	TLRM	$p = 0.0002$	$p = 0.0139$
External validation cohort 3	TLRM	$p = 0.0133$	$p = 0.0060$
Whole validation data	TLRM	$p < 0.0005$	$p < 0.0005$
**IDI**			
Dataset	Model 1 / Model 2	Clinical model	TLS-LW
Internal validation cohort	TLRM	0.0443 ($p < 0.0005$)	0.0155 ($p = 0.0070$)
External validation cohort 1	TLRM	0.0367 ($p = 0.0002$)	0.0327 ($p < 0.0005$)
External validation cohort 2	TLRM	0.0452 ($p = 0.0083$)	0.0109 ($p = 0.2371$)
External validation cohort 3	TLRM	0.0484 ($p = 0.0051$)	0.0188 ($p = 0.0141$)
Whole validation data	TLRM	0.0385 ($p < 0.0005$)	0.0222 ($p < 0.0005$)

Notes: IDI, integrated discrimination improvement; TLRM, transfer learning radiomics model; TLS-LW, transfer learning signature based on lung whole slide images.

Figure S1

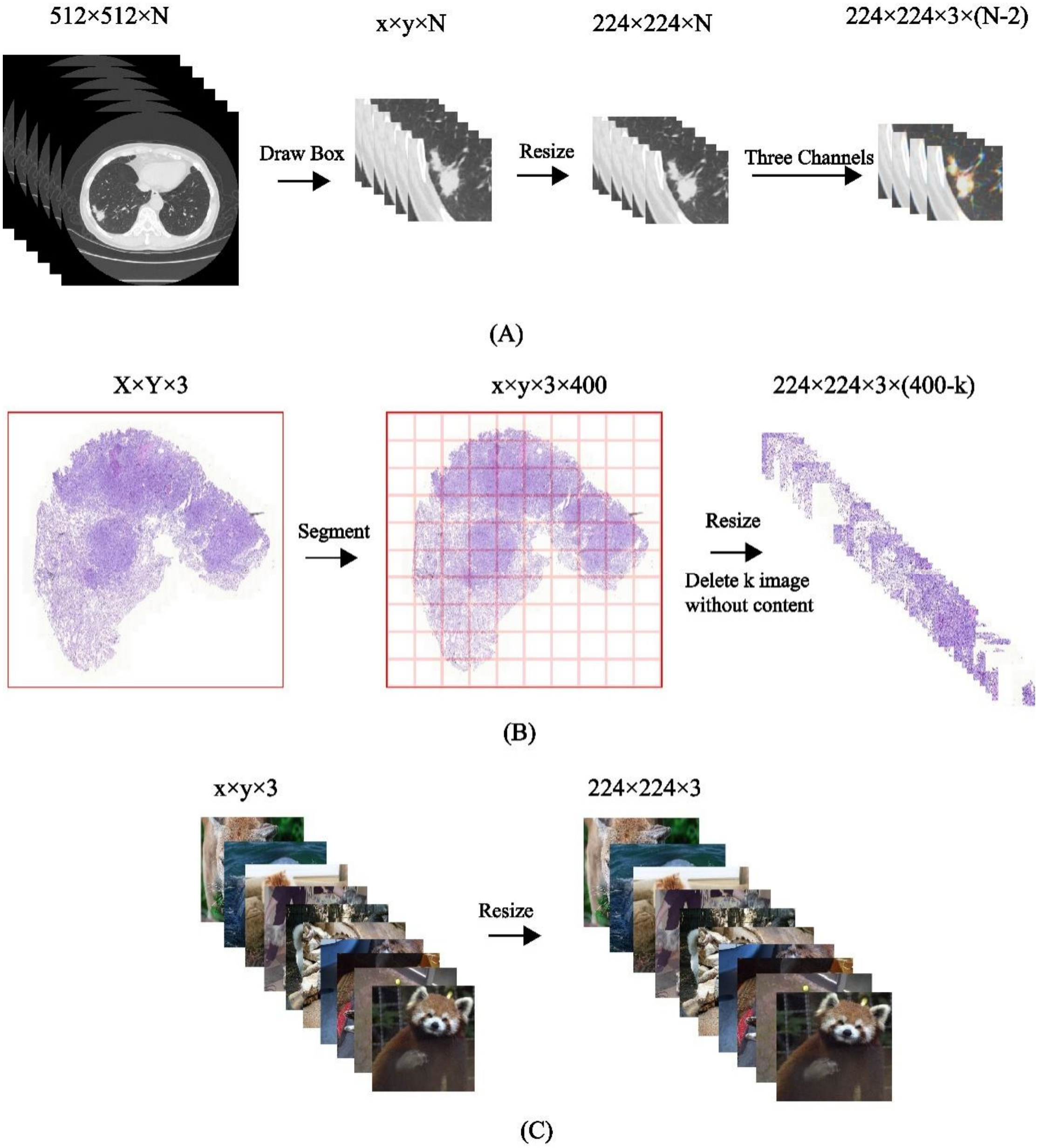

The pre-processing processes of CT images (A), whole slide images (B) and ImageNet (C), respectively.

Notes: N is the number of transverse-images of a patient with SPSNs; CT, computed tomography; SPSNs, solitary pulmonary solid nodules.

Figure S2

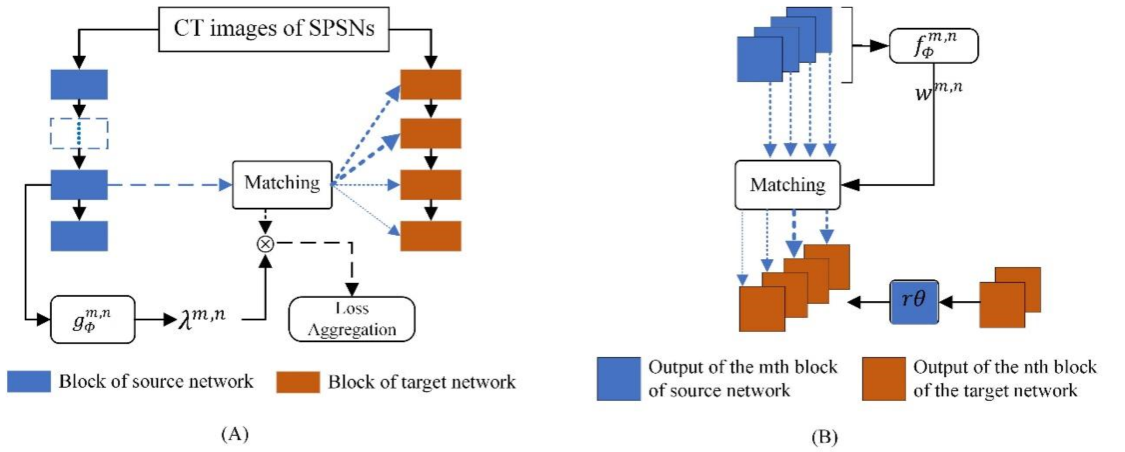

The structure of source domain feature selection networks. The source domain feature selection networks were parameterized by ϕ and were learned for selective knowledge transfer via meta-learning. The dashed lines indicate flows of tensors such as feature maps, and solid lines denote feature matching. (a) $g_{\phi}^{m,n}$ outputs weights of matching pairs $\lambda^{m,n}$ between the mth and nth blocks of the source and target network, respectively, and (b) $f_{\phi}^{m,n}$ outputs weights for each channel.

Notes: CT, computed tomography.

Figure S3

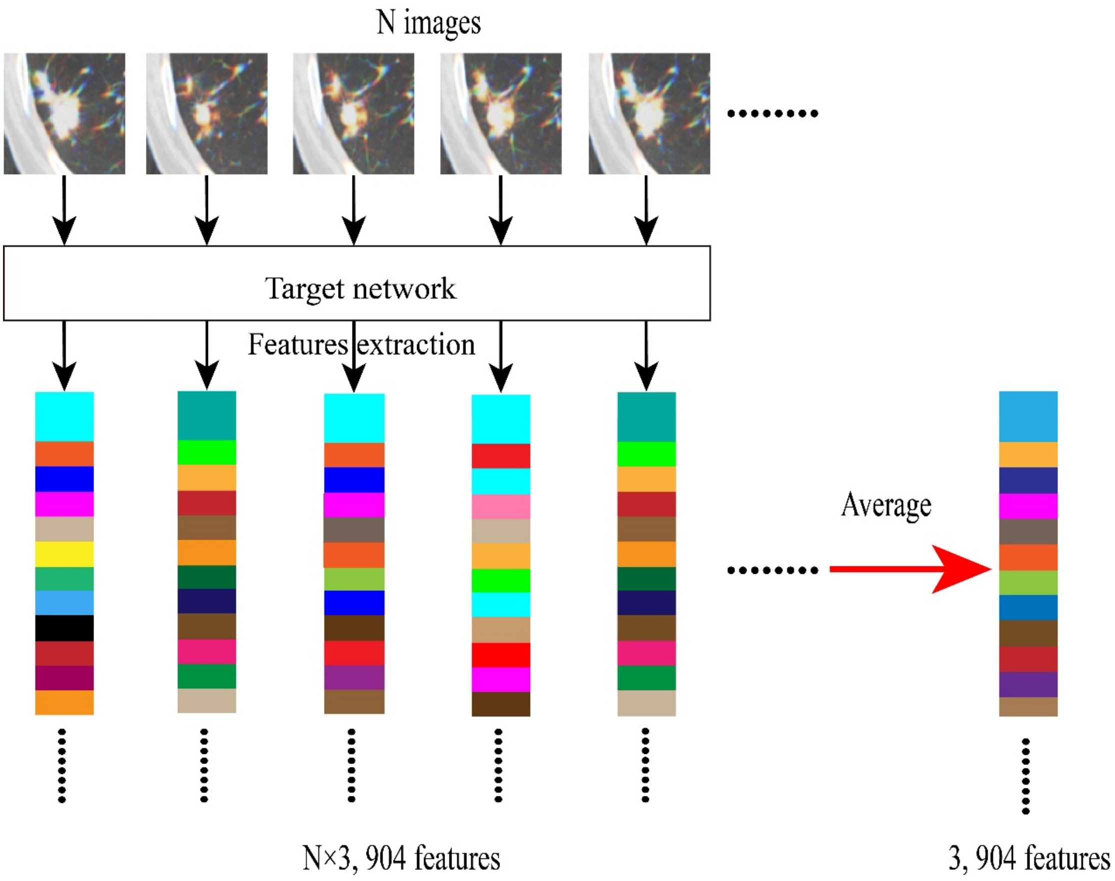

Processes of transfer learning feature extraction for each patient. A single patient contains N preprocessed tumor images. Since the target network has 3, 904 convolution kernels, 3, 904 features were extracted per image. The N features from the same filter were averaged. Thus, every single patient corresponded a group feature that included 3,904 features.

Figure S4

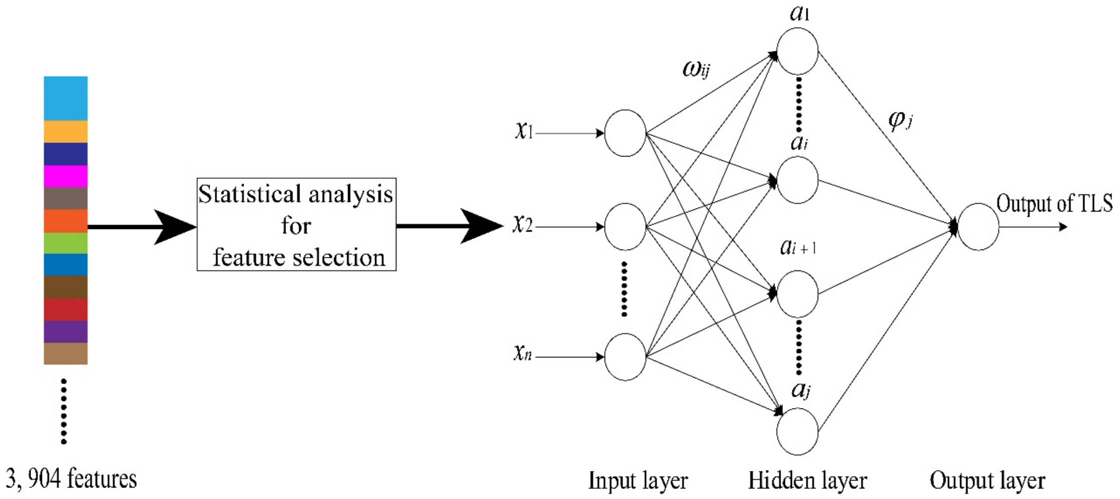

Feature selection and structure of sparse Bayes-based extreme learning machine. Notes: TLS, transfer learning signature.

Figure S5

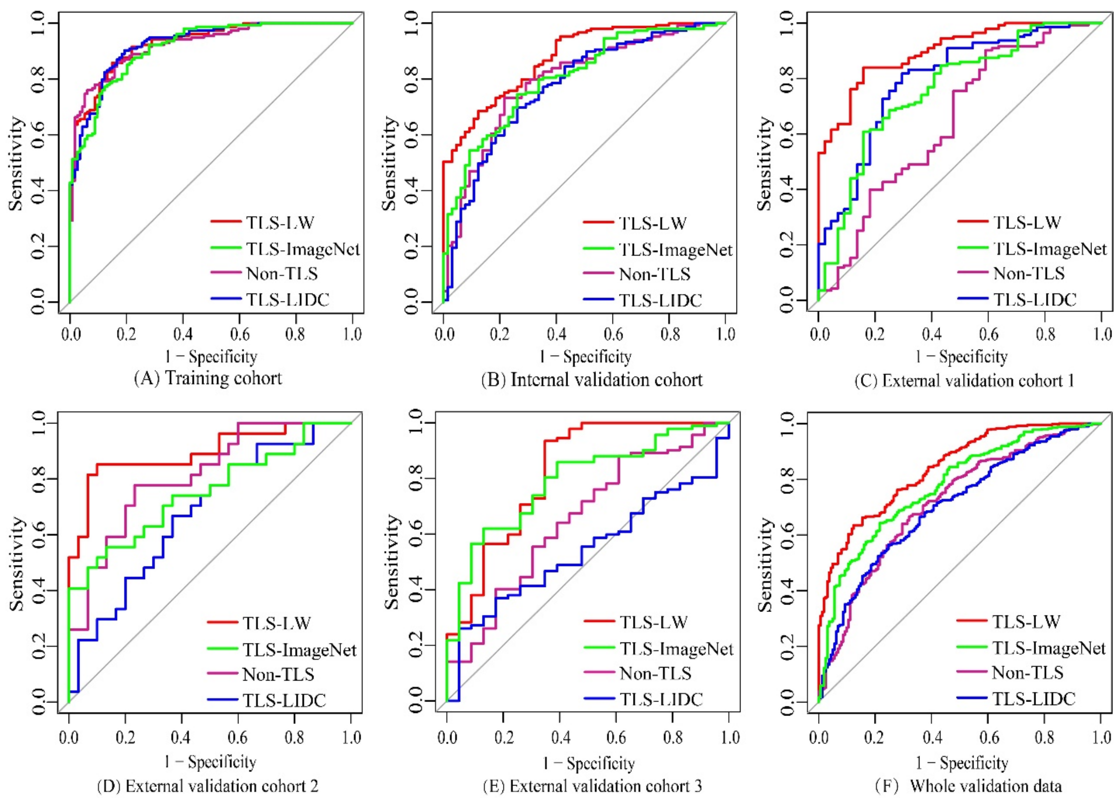

ROC curves of Non-TLS, TLS-ImageNet, TLS-LIDC, and TLS-LW in the (A) training cohorts, (B) internal validation cohorts, (C) external validation cohorts1, (D) external validation cohorts 2, (E) external validation cohorts 3, and (F) whole validation data 3.

Notes: Non-TLS, non-transfer learning signature; TLS-LIDC, transfer learning signature based on LIDC; TLS-ImageNet, transfer learning signature based on ImageNet; TLS-LW, transfer learning signature based on lung whole slide images.

Figure S6

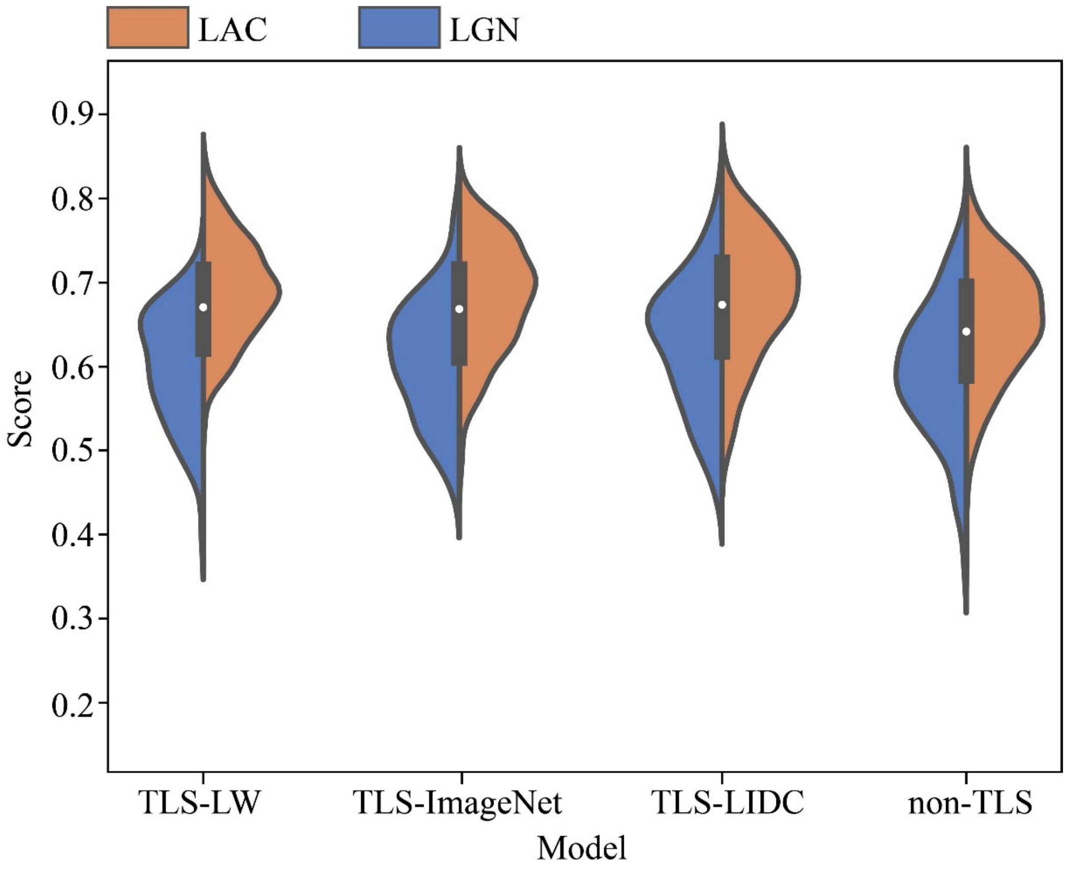

The score of TLS-LW, TLS-ImageNet, TLS-LIDC and non-TLS in the whole validation data.

Notes: TLS-LIDC, transfer learning signature based on LIDC; TLS-ImageNet, transfer learning signature based on ImageNet; TLS-LW, transfer learning signature based on lung whole slide images; Non-TLS, non-transfer learning signature; LAC, lung adenocarcinoma; LGN, lung granulomatous nodule.

Figure S7

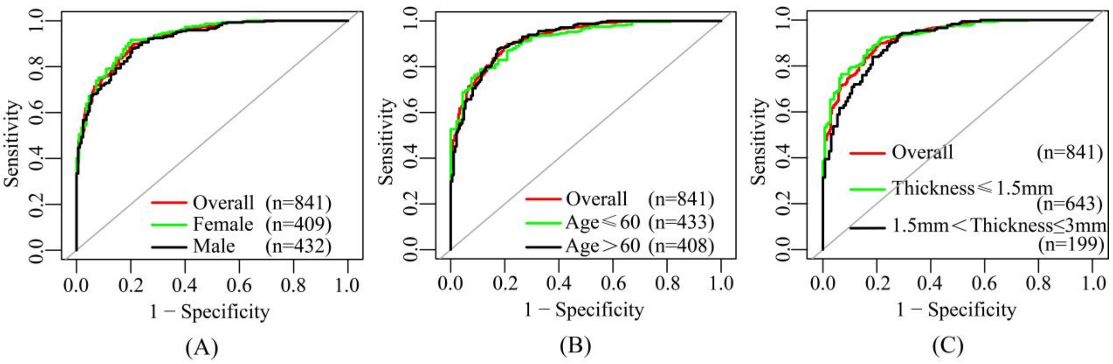

Stratified analysis of (A) gender, (B) age, and (C) CT slice thickness.
